# Effect of Lifestyle Modification Through Web-Based Telerehabilitation Monitoring Combined With Supervised Sensorimotor Training After Total Knee Arthroplasty: Randomized Controlled Trial

**DOI:** 10.2196/64643

**Published:** 2025-10-02

**Authors:** Samreen Sadiq, Rabiya Noor, Rizwan Akram

**Affiliations:** 1 Department of Physical Therapy, Riphah College of Rehabilitation and Allied Health Sciences, Riphah International University, 25 Raza Saeed Rd, Bhabra Block M Gulberg III, Lahore, 54660, Pakistan, 92 3344355660; 2 Department of Orthopedic and Spine Center, Ghurki Trust Teaching Hospital, Lahore, Pakistan

**Keywords:** prescribed home-based program, supervised training, total knee replacement, telerehabilitation, web based portal, monitoring, lifestyle modification, total knee arthroplasty, knee, knee osteoarthritis, osteoarthritis, low back pain, Asia, quality of life, digital rehabilitation, rehabilitation, single-center, randomized controlled trial, mobile health, telehealth, digital health

## Abstract

**Background:**

Total knee arthroplasty (TKA) is commonly performed to manage end-stage knee osteoarthritis, yet postsurgical recovery varies significantly among patients. Lifestyle modification and rehabilitation interventions play a critical role in optimizing outcomes. While telerehabilitation has shown promise in enhancing accessibility and compliance, its role in supporting lifestyle behavior change alongside supervised sensorimotor training remains underexplored.

**Objective:**

This study aimed to evaluate the effects of a home-based lifestyle modification program delivered through web-based telerehabilitation monitoring in addition to supervised sensorimotor training, in improving physical function, pain, balance, quality of life (QOL), and adherence in patients undergoing TKA.

**Methods:**

A single-blinded randomized controlled trial was conducted among 52 participants undergoing primary TKA, who were randomly assigned to either the intervention group (IG) (supervised sensorimotor training plus a telerehabilitation-supported lifestyle modification program) or the control group (CG) (supervised sensorimotor training alone and a traditional home exercise plan). The intervention lasted 22 weeks, and participants were assessed at baseline (presurgery), 14 weeks, and 22 weeks postsurgery. Outcome measures included joint position sense (JPS), musculoskeletal ultrasound of the rectus femoris muscle, Berg Balance Scale, knee function using the Knee Injury and Osteoarthritis Outcome Score, and QOL via EuroQol 5-dimension 5-level questionnaire.

**Results:**

Significant improvements were observed in the IG across all outcomes compared with the CG. Notably, the IG showed greater improvements in musculoskeletal ultrasound thickness. JPS showed superior accuracy in the experimental group (baseline [3.2 degrees] to 22 wk postsurgery [0.05 degrees]) compared with the CG (baseline [3.1 degrees] to 22 wk postsurgery [1.8 degrees]), with significant improvements noted (*P*=.001, Cohen *d*=3.1 vs 0.7), Knee Injury and Osteoarthritis Outcome Score subscales (pain, symptoms, activities of daily living, sport, and QOL), and JPS (mean absolute error 0.05 vs 1.8 degrees). Berg Balance Scale demonstrated significant gains in balance for the experimental group (baseline [34] to 22 wk postsurgery [53]) relative to the CG (baseline [37] to 22 wk postsurgery [48]), with substantial differences observed (*P*=.001, Cohen *d*=1.8 vs 0.4). The EuroQol 5-dimension 5-level questionnaire health-related QOL scores were markedly higher for the experimental group (baseline [45.4] to 22 wk postsurgery [88.1]) compared with the CG (baseline [42.8] to 22 wk postsurgery [70.9]), indicating substantial gains in overall health status (*P*=.001, Cohen *d*=2.4 vs 1.3). The IG also reported higher compliance, with 81.8% (18/22) achieving over 90% adherence compared with 68.18% (15/22) in the CG.

**Conclusions:**

Home-based lifestyle modification program through telerehabilitation monitoring significantly improved functional and patient-reported outcomes in individuals following TKA. These findings support the integration of lifestyle modification programs through telerehabilitation monitoring into post-TKA recovery pathways to optimize rehabilitation outcomes.

## Introduction

Knee osteoarthritis (OA) and low back pain are prevalent rheumatic disorders in Asian regions, posing significant challenges to patients and health care systems [1]. Total Knee Arthroplasty (TKA) is widely recognized as the gold standard management strategy for relieving symptoms associated with degenerative knee arthritis [2]. Rehabilitation following TKA plays a crucial role in improving functional performance and quality of life (QOL) for individuals in this population. However, access to rehabilitation services, health care facilities, and follow-up appointments can be limited, particularly in regions with resource constraints or remote areas [3]. Telerehabilitation, a branch of telehealth, presents a potential solution by using technology to deliver monitoring and rehabilitation services to patients from a distance, ensuring adequate follow-up and support [4].

One important aspect of post-TKA rehabilitation is sensorimotor training, which aims to improve neuromuscular control, balance, and coordination to restore functional abilities and reduce the risk of falls. Sensorimotor training focuses on optimizing central nervous system function and promoting appropriate muscular firing patterns for joint stability [5]. By incorporating sensorimotor training into telerehabilitation programs, patients can receive personalized exercises and real-time feedback from health care professionals, even when they are at home. Another crucial component missing from many post-TKA rehabilitation programs is lifestyle modification. Lifestyle modifications encompass a range of interventions that focus on promoting healthy behaviors and habits to improve overall well-being [6]. In the context of post-TKA rehabilitation, incorporating lifestyle modification programs into telerehabilitation monitoring can have several benefits for patients. Providing patients with educational resources and information about lifestyle factors that can impact their joint health is essential. Telerehabilitation can offer interactive educational materials, including videos, articles, and digital sessions, to educate patients about the importance of maintaining a healthy lifestyle. Topics may include proper nutrition, weight management, joint protection techniques, and strategies to reduce joint stress in daily activities [7]. Regular exercise is a cornerstone of lifestyle modification and plays a crucial role in managing joint health after TKA. Telerehabilitation platforms can provide guided exercise programs tailored to individual patients, including specific exercises targeting muscle strengthening, flexibility, and cardiovascular fitness. Through video demonstrations and remote monitoring, health care professionals can ensure that patients perform exercises correctly and modify the program as needed to accommodate their progress [8]. Nutrition plays a vital role in joint health and overall well-being. Telerehabilitation can integrate dietary guidance by providing personalized nutrition plans and recommendations [9].

Excessive body weight can contribute to increased joint stress and deteriorate the outcomes of TKA. Telerehabilitation monitoring can include weight management programs that track patients’ weight, provide nutritional guidance, and offer support in setting realistic weight loss goals [10]. Lifestyle modifications through telerehabilitation can address behavior change techniques to help patients adopt and maintain healthy habits. This may include setting goals, self-monitoring techniques, problem-solving strategies, and relapse prevention [11].

Integrating lifestyle modification programs into telerehabilitation monitoring for post-TKA patients can have a comprehensive impact on their overall outcomes [12]. By addressing education, exercise, dietary interventions, weight management, and behavior change, patients can experience improved joint health, reduced joint stress, better functional outcomes, enhanced QOL, and increased long-term success of the TKA procedure. Furthermore, telerehabilitation provides a convenient and accessible platform for patients to receive ongoing support and guidance, promoting adherence to lifestyle modifications even after formal rehabilitation sessions are completed [13]. Patients can access resources remotely, receive reminders and encouragement, and engage in digital consultations to address any concerns or challenges they may face. While digital rehabilitation solutions are well-established in developed nations, there is a pressing need to introduce technological advancements in countries like Pakistan. Telerehabilitation, if incorporated into standard therapy, has the potential to benefit the community by reducing dependence on limited human resources while ensuring better clinical outcomes [14]. By embracing telerehabilitation, health care providers in Pakistan can overcome geographical barriers, increase access to rehabilitation services, and enhance patient care in a cost-effective manner.

TKA is commonly followed by structured rehabilitation programs to restore joint function and QOL. Though various rehabilitation protocols, including supervised sensorimotor training, have been extensively studied, a vital component termed lifestyle modification in the postoperative period remains principally overlooked. Current literature lacks the incorporation of home-based lifestyle interventions that address patients’ daily habits, activity levels, and long-term behavioral changes, which are crucial for continual recovery and enhanced outcomes after TKA. This study aims to fill this critical gap by evaluating the combined effect of supervised sensorimotor training and structured lifestyle modification, delivered through telerehabilitation monitoring. The significance of this study lies in its unique approach to combine digitally monitored physical rehabilitation with behavior-driven home-based strategies, targeting not only physical but also the psychosocial and practical facets of recovery. This integrated model could offer a more holistic and sustainable rehabilitation pathway, particularly in underserved settings.

Therefore, the main aim of our study was to evaluate the effects of lifestyle modifications through telerehabilitation monitoring combined with supervised sensorimotor training on various outcomes, including joint position sense (JPS), balance, muscle architecture, knee joint function, and QOL among patients with TKA.

## Methods

### Study Setting and Design

We conducted a single-centered randomized controlled trial with parallel groups between 2021 and 2023 aimed to compare the effect of a home-based lifestyle modification program delivered through telerehabilitation monitoring, combined with supervised sensorimotor training for patients undergoing elective TKA for unilateral OA. This study received approval from the Research and Ethics Committee at Riphah College of Rehabilitation and Allied Health Sciences, Riphah International University, Lahore, Pakistan (REC/RCR&AHS/2033) and was conducted in accordance with the Declaration of Helsinki [15]. The protocol of the study has been officially registered with the clinical study identifier NCT05018494 in the ClinicalTrials.gov Protocols Registration and Results System on May 8, 2021. The participating center in this study was the Physical Therapy department of Ghurki Trust Teaching Hospital, Lahore, which was following the rehabilitation protocol for patients post-knee replacement.

### Sample Size

Sample size calculation was conducted using G*Power (version 3.1.9.4; Heinrich Heine University Düsseldorf) based on the Knee Injury and Osteoarthritis Outcome Score (KOOS) pain dimension, which had demonstrated a large effect size (Cohen *d*=2.0) in previous literature [16], demonstrating substantial differences between the intervention groups (IGs). To ensure adequate power to detect significant changes, the calculation was performed with a significance level (*α*) of .05 (2-tailed) and a statistical power of 80%. The pooled SD for the KOOS pain dimension was calculated as 11.26, and the mean difference between the experimental and control groups (CGs) was 13.4 points. Based on these parameters, the required sample size was calculated to be 26 participants per group, resulting in a total sample size of 52 participants. This sample size was sufficient to detect meaningful differences in pain outcomes between the groups. Moreover, considering potential participant dropout rates of approximately 10%‐15%**,** a total of 52 participants were estimated to be adequate to maintain the study’s statistical power.

### Study Participants

Eligibility for the study was assessed for all patients who had received an anesthesiologist’s approval and were already scheduled for surgery. Patients were invited to participate in the study, and detailed oral and written explanations were provided about the trial. Those willing to participate were required to sign a written informed consent form. Prior to admission to the hospital, patients completed preoperative outcome assessment questionnaires, including the knee joint function, Berg Balance Scale (BBS), QOL questionnaire, along with objective measures of JPS and cross-sectional area of rectus femoris (RF) through diagnostic musculoskeletal ultrasound (MSK-US).

The inclusion criteria were (1) patients who were men or women, (2) had undergone primary TKA surgery, (3) had the ability to walk (with a walking aid or unaided), (4) were aged between 45 and 75 years, (5) had active knee flexion of 80° and active knee extension of −10° upon discharge, and (6) had the availability of a smartphone and internet service in the residing area.

The exclusion criteria were (1) the presence of health-related medical conditions that could interfere with tests or the rehabilitation program, (2) neurological conditions and vestibular disorders that might affect balance, (3) inability to attend rehabilitation services, revised knee arthroplasty, and (4) blindness and any condition incompatible with 30 minutes of light to moderate physical activity.

### Randomization, Group Allocation, and Blinding

Participants were randomized to groups on a 1:1 basis through computer-generated randomization performed by an independent statistician. Following the initial assessment, a volunteer rehabilitation house officer distributed envelopes based on the randomization sequence. These envelopes, which were consecutively numbered, sealed, and opaque, were stored in a secure location accessible only to the unblinded lead researcher-physiotherapist (SS). This physiotherapist (SS) then scheduled the participants’ training session to introduce the web-based rehabilitation lifestyle modification program. Participants reviewed and signed the informed consent form, which adhered to the guidelines of the Helsinki Declaration and outlined the study’s objectives.

### Intervention and Control Group

**Figure 1.**
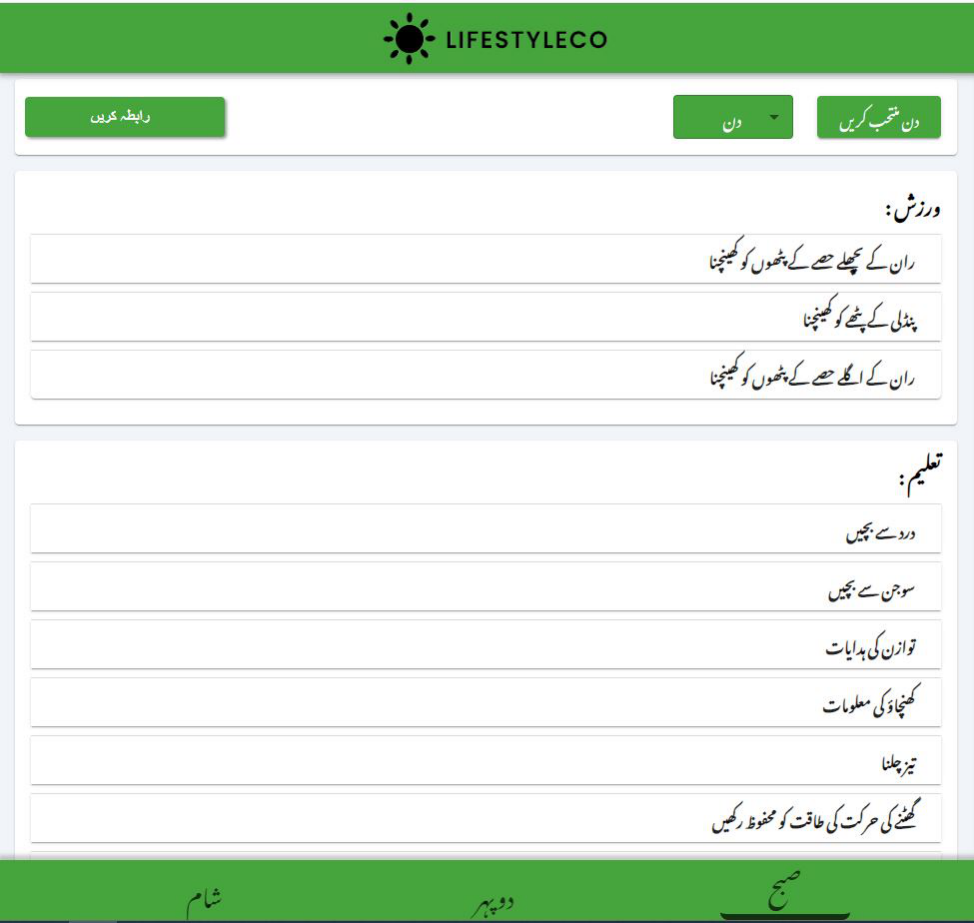
Digital lifestyle modification process.

**Figure 2.**
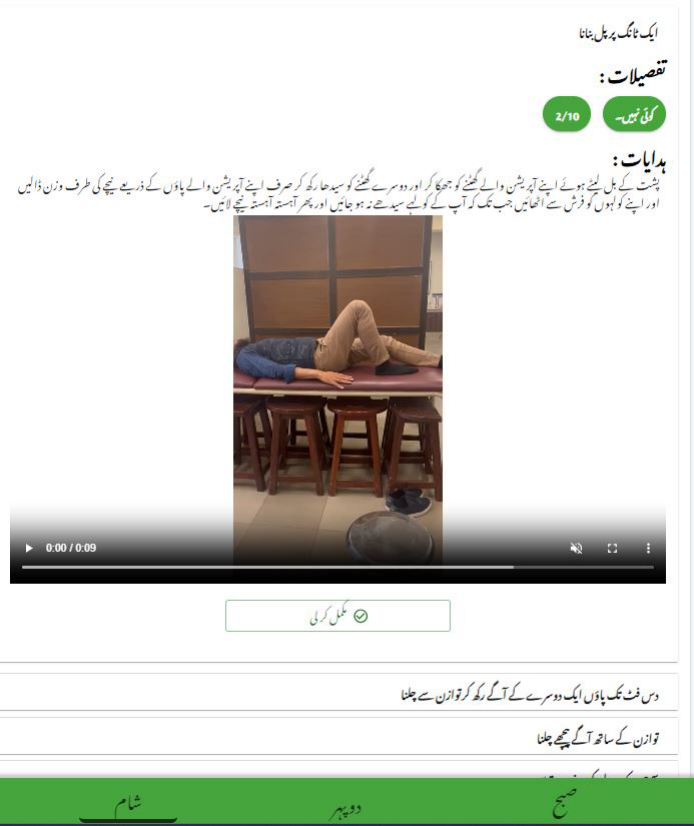
Exercise with instructional videos and Urdu guidance.

**Figure 3.**
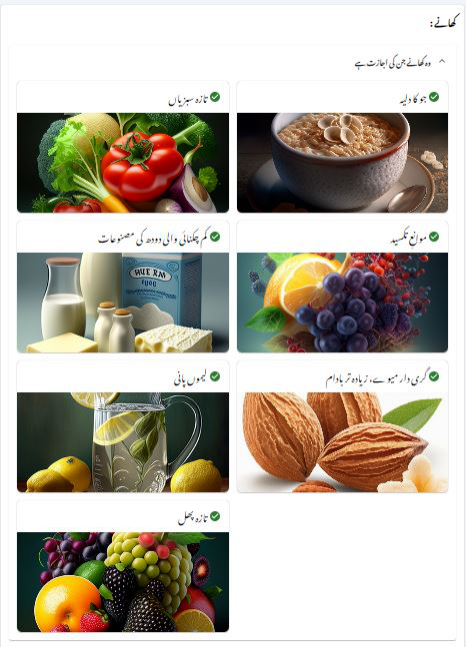
Diet plan view.

Once included in the study, post 2 weeks of knee replacement, patients were randomized into either the IG or the CG. The randomization process was blinded to patients. Patients in the IG and CG received one-on-one 12 weeks of supervised strengthening exercises, neuromuscular electrical stimulation, and sensorimotor training, 3 times a week for 45 minutes to learn strength and sensory-motor exercises. Patients who received any rehabilitation limitations from the surgeon were excluded from the study. At the time of discharge, patients in IG received a web portal link that could be viewed on any smartphone or device with a complete manual on lifestyle modification, constituting 3 sections (Figure 1). The first section included exercises comprising videos of morning, afternoon, and evening, along with clear instructions in the local Urdu language for better understanding (Figure 2). The second section was about a daily diet plan of morning, afternoon, mid-evening, and night meals, considering the optimal caloric balance (Figure 3). The third section was the education section, giving instruction points about safety and daily activities. The CG patients received standard written instructions commonly prescribed as home exercises with oral guidance.

Regular messages and phone calls every week were conducted to follow up with all patients, addressing any possible complications, completion of the training diary, and exercise clarity. The patients had scheduled hospital visits at 3 and 5 months. During the follow-up visit, the physical therapist performed assessments, and patients were asked to fill out questionnaires. Postoperative cross-sectional area of the RF muscle was taken to compare the muscle architecture presurgery, after sensorimotor training, and post home-based exercises. Three readings were taken for all the measures (presurgery, 12 weeks after sensorimotor training, and a further 8 weeks after home-based lifestyle modification implementation). At follow-up, the physiotherapist inquired about any adverse effects or reasons for training cessation from the patients. This comprehensive study design allowed us to assess the effectiveness, safety, and long-term outcomes of a home-based lifestyle modification manual delivered through telerehabilitation monitoring compared with the standard home-based exercises for patients undergoing elective TKA.

Participants maintained compliance through their portal by checking off their exercise, educational, and dietary activities. Any adverse events were promptly reported to the therapist via the WhatsApp button available on the portal. Exercise assurance videos were also sent through WhatsApp for additional guidance. They recorded each exercise performed daily, made notes about any additional sports activities such as walking or stationary bicycling, documented any adverse effects experienced, and provided reasons for skipping any program element if applicable. Participants also used the Visual Analog Scale (VAS) to record their pain levels throughout the day, before and after training sessions, and at night.

### Standard Rehabilitation

All patients received standard physiotherapy care during their hospitalization. This began on the day of surgery and included mobilization using a walking aid (typically 2 crutches, occasionally a walker), exercises to prevent deep vein thrombosis, lower limb range of motion exercises, and an isometric strengthening program. Patients were advised to perform these exercises twice daily, aiming for approximately 10 repetitions of each exercise, and to walk as much as possible. The exercises were conducted in various positions, including supine, on the healthy side, on the abdomen, sitting, and standing (eg, buttock squeezes, leg sliding motions, straight leg raises, bridges, and postural exercises).

The treatment protocol involved a structured regimen starting with 2 weeks of hospital-based bedside and preparatory physiotherapy. This was followed by 12 weeks of supervised strengthening exercises, neuromuscular electrical stimulation, and sensorimotor training, conducted 3 times a week for 45 minutes each session. Additionally, an 8-week lifestyle modification program encompassing education, exercise, and diet was provided to the IG. Common treatments consisted of strengthening exercises, neuromuscular electrical stimulation, and sensorimotor training, all carried out over a 12-week period with sessions 3 times a week for 45 minutes. Each session included routine elements such as warm-up activities involving range-of-motion exercises, stretching, or flexibility exercises; mobility exercises for the lower extremities; and strengthening exercises, including isometric and concentric exercises for the knee quadriceps, hamstrings, and hip abductors. Functional, task-oriented exercises such as stair climbing, squats, stationary cycling, treadmill walking, or regular walking were also part of the regimen, followed by cool-down activities. Concentric mode strengthening exercises were performed using a medium-resistance elastic theraband provided to all patients. The primary focus of the exercises was enhancing sensorimotor functioning. Innovative agility and perturbation training techniques were incorporated, such as side-stepping, backward stepping, and using irregular foam laminations to offer targeted sensorimotor stimulation over unstable surfaces [17]. Exercise challenges and progression were achieved using regular pillows to simulate unstable surfaces, plastic cups to create obstacles, and strategies such as transitioning from bipedal to monopedal stance and from eyes open to eyes closed to increase the difficulty of maintaining or achieving balance. Patients in the CG were given written instructions, handouts with pictorial exercises, and a portal login that provides access to exercise videos along with a checklist to mark daily exercise compliance.

### Prescribed Digital Home-Based Lifestyle Modification Program

**Figure 4.**
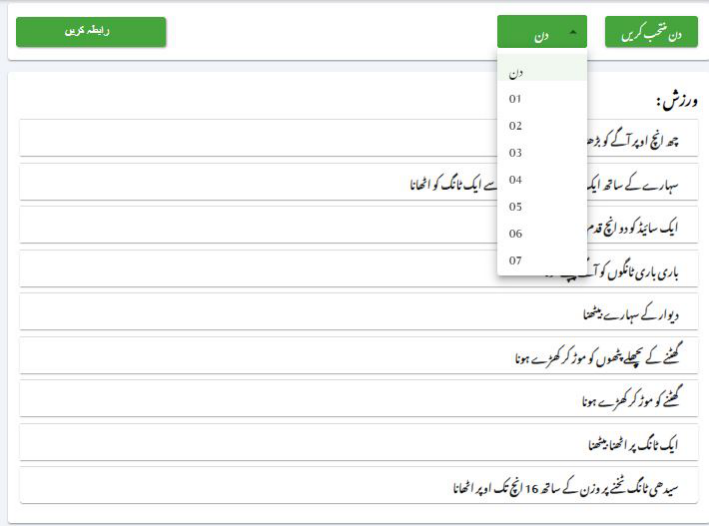
Select day and time.

Before discharge, physiotherapists instructed patients in the IG on specialized strength and sensorimotor exercises using various exercise aids along with educational points and a diet plan. These patients also received a web portal login containing exercise videos, written instructions, a diet plan with pictorial representation, and a checklist to track their daily lifestyle routine (Figure 4). The new training protocol included exercises designed to enhance hip stability and decrease local stress on the knee prosthesis. Safety considerations regarding knee, hip, and lumbar spine load; fall prevention; and prosthesis dislocation were integral to the training design [18]. The program encompassed hip and knee muscle strengthening exercises (emphasizing the abductors), hip and pelvic stabilization exercises, ankle and knee muscle strengthening exercises (to better dissipate impact forces and control femoral internal rotation), and trunk muscle strengthening exercises (to stabilize the pelvis and lumbar spine, thereby reducing local loads). The exercises were designed to be simple and easy to understand, with home exercise videos that are straightforward to follow. Additionally, the training program avoided the need for expensive equipment. Focusing on education, physical activity, dietary modifications, weight management, and behavioral changes might lead to better joint health, decreased joint stress, improved functional outcomes, enhanced QOL, and increased long-term success of the TKA procedure. The detailed lifestyle modification plan was attached in the Multimedia Appendix 1.

### Outcome Measures

The primary objective of the study was to evaluate the effectiveness of the chosen protocol, which was assessed through JPS, muscle thickness of the RF, and balance. Secondary outcomes included knee function, and QOL was assessed through patient questionnaires, as well as consistent reporting of any adverse event.

### Knee Joint Position Sense

Knee JPS was assessed using a reliable and validated method, incorporating measurements of joint angles through digital photography and nonreflective markers, supported by specialized software. The JPS assessment was performed in a quiet, isolated room. Patients were seated in a high sitting position on a treatment plinth, and testing was conducted on the operated knee. Four square nonreflective markers, each 4 cm in diameter, were placed at specific anatomical landmarks on the lateral side of the limb: First, proximal to a quarter of the distance along a line from the greater trochanter to the lateral joint line of the knee, second, over the neck of the fibula, third over the proximal part of the lateral malleolus, and fourth on the iliotibial tract adjacent to the superior border of the patella. With eyes closed, the patient’s knee was passively moved by the examiner to one of 2 target reference angles, 30° or 60° of knee extension, and held in that position for approximately 5 seconds to allow the patient to internalize the joint position. The limb was then returned to the resting position for 7 seconds. The patient was subsequently asked to actively reproduce the same knee angle. Each angle was tested 3 times, and the average value of the reproduced angles was calculated. A side-view photograph was taken for each trial using an Apple iPhone 14 Pro Max (48 MP camera), positioned 185 cm from the side of the patient and 65 cm above the floor. The angles formed by the markers were analyzed using Paint software by drawing lines between points to measure joint angles, and final values, including the absolute angular error, were calculated and documented in Microsoft Excel. Absolute error was defined as the absolute difference between the target and perceived angles for each of the 3 trials at specified angles for each limb of each participant. Relative error indicates the variability of errors between trials, reflecting the consistency of proprioceptive performance [19].

### Muscle Thickness of Rectus Femoris

MSK-US was used using a curvilinear probe operating at frequencies ranging from 7.1 to 14 MHz, along with a 55 mm linear probe. The probe was positioned ventrally in the transverse plane, perpendicular to the skin over the RF muscle. The RF muscle’s location was marked at a point 3/5 of the distance from the anterior superior iliac spine to the superior border of the patella, ensuring that the entire cross-section of the RF is visible within a single field for all patients [20].

Imaging was conducted with patients seated. Gentle contraction and relaxation maneuvers were used to enhance visualization of muscle septa before capturing images. Cross-sectional area, muscle thickness, and width were measured at this specific muscle point, with images taken both in complete relaxation and during contraction.

### Balance

The original 14-item BBS was used to evaluate balance in older individuals. This scale has been validated, proven reliable, and shown to be responsive in assessing balance. It comprises 14 straightforward balance tasks, each scored on a 5-point ordinal scale (0, 1, 2, 3, and 4), with a maximum possible score of 56. Higher scores on the BBS indicate better balance. This assessment tool is widely used clinically, with all tasks focusing on balance, and has previously been used to evaluate balance in patients recovering from hip fractures and knee arthritis [21].

### Knee Function

Knee function was assessed using the KOOS. Originating from the WOMAC Osteoarthritis Index in 1995, the KOOS is a reliable instrument for evaluating symptoms and function in individuals with knee injuries or OA. This self-administered questionnaire takes approximately 10 minutes to complete and encompasses 5 subscales: pain, symptoms other than pain, function in activities of daily living (ADL), function in sports and recreation (Sport/Rec), and knee-related QOL. Scores are reported as percentages ranging from 0 to 100. This comprehensive tool enables both short-term and long-term assessment of various knee conditions, including OA. The Urdu version of the KOOS was used for this evaluation [22].

### Quality of Life

The EuroQol 5-dimension 5-level questionnaire (EQ-5D-5L) was used to assess health-related quality of life (HRQOL). Its conceptual framework takes a holistic approach to health, encompassing not only the medical definition but also the essential aspects of independent physical, emotional, and social functioning. The EQ-5D-5L considers health as a composite of positive elements (well-being) and negative aspects [23]. It comprises a questionnaire and a VAS (EuroQol VAS), where the EuroQol VAS allows individuals to rate their own current overall health status using a VAS [24]. This scale enables continuous monitoring of health changes over time. The self-assessment questionnaire involves the participant describing their current health status across 5 dimensions: mobility, self-care, usual activities, pain/discomfort, and anxiety/depression. Participants are asked to assess their level of function in each dimension as severe, moderate, or none.

### Telerehabilitation Monitoring

Participants maintained compliance through their portal by checking off their exercise, educational, and dietary activities. Any adverse events were promptly reported to the therapist via the WhatsApp button available on the portal. Exercise assurance videos were also sent through WhatsApp for additional guidance. They recorded each exercise performed daily, made notes about any additional sports activities such as walking or stationary bicycling, documented any adverse effects experienced, and provided reasons for skipping any program element if applicable. Participants also used the VAS to record their pain levels throughout the day, before and after training sessions, and at night.

All of this information was analyzed to evaluate patient satisfaction, assess the feasibility of the protocols, and ensure their safety.

### Statistical Analysis

All data was collected and recorded in hard copy format by the primary author, who bears responsibility for maintaining data confidentiality. Check-up forms were printed and completed preoperatively and at each subsequent check-up by the physiotherapist and orthopedic surgeon. Patients also filled out printed questionnaires during these sessions. The author entered the anonymized data into tables for statistical analysis. Statistical processing was conducted using IBM SPSS Statistics (version 25.0; IBM Corp).

An intention-to-treat analysis was performed to mitigate potential biases arising from missing data in randomized controlled trials. This analytic approach involved analyzing participants based on their original randomized treatment assignment, regardless of whether they completed the treatment as intended. Sensitivity analyses were conducted and reported to address any missing data [25].

Descriptive statistics were presented as mean (SD), and intergroup differences in baseline characteristics will be assessed using an independent *t* test. The normality of the sampling distribution was evaluated using the Shapiro-Wilk W-test for inferential analysis [26]. Assumptions of sphericity (Mauchly test) and homogeneity of variances (Levene test) were checked prior to analysis. Repeated Measures ANOVA was used to compare mean score changes both between and within groups to evaluate the effects of the intervention. The significance level for all analyses was set at *P*<.050.

Mixed effects ANOVA assessed the impact of Group (experimental and control), Time (baseline, 14 wk, and 22 wk), and their interaction on each dependent variable. Effect sizes were computed using partial eta squared (η²p), with values between 0.1 and 0.24 indicating a moderate effect, values from 0.25 to 0.36 indicating a medium effect, and values of 0.37 or higher indicating a large effect [27]. To control for potential inflation of Type I error due to multiple outcome measures, the Holm-Bonferroni correction and Benjamini-Hochberg (FDR) methods were applied to *P* values of all outcomes.

### Ethical Considerations

This study was approved by the Research and Ethics Committee at Riphah College of Rehabilitation and Allied Health Sciences, Riphah International University, Lahore, Pakistan (REC/RCR&AHS/2033), and was conducted in accordance with the ethical principles outlined in the Declaration of Helsinki. The study protocol was officially registered with ClinicalTrials.gov (Identifier: NCT05018494) on May 8, 2021. Written informed consent was obtained from all participants prior to their inclusion in the study. Participants were fully informed about the study’s purpose, procedures, potential risks, and benefits. To maintain privacy and confidentiality, all collected data were anonymized and securely stored, with access to the telerehabilitation platform restricted to authorized personnel through password-protected accounts. No financial compensation was provided for participation, and all rehabilitation services, materials, portal logins, and digital access were offered free of charge.

## Results

**Figure 5.**
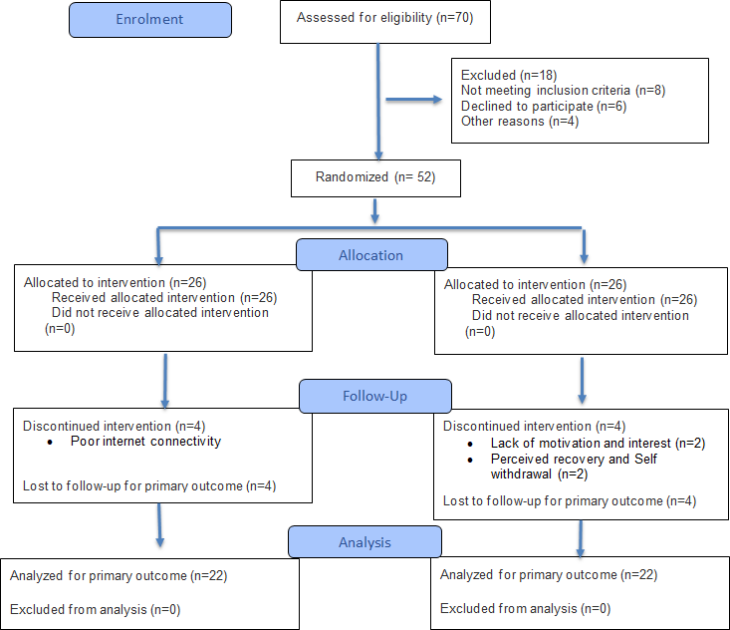
Consort flow diagram.

Out of 70 participants initially assessed for eligibility, 52 were randomly allocated—26 to the IG and 26 to the standard care group. However, due to participant dropouts, the final assessment comprised 22 individuals from the experimental group and 22 from the CG. The different stages of the study were outlined in the CONSORT flowchart presented in Figure 5. The data in Table 1 illustrate the baseline characteristics of both the interventional group (n=22) and the CG (n=22). Statistically significant differences were not observed between the two groups (*P*>.50), suggesting that both groups were well-aligned at baseline.

**Table 1.** Participants’ demographic characteristics.

Characteristics	Experimental group (n=22)	Control group (n=22)	*P* value
Age (years), mean (SD)	64.1 (5.4)	63.5 (5.5)	.63
Body mass (kg), mean (SD)	74.5 (4.8)	75.0 (5.5)	.98
Height (m), mean (SD)	1.68 (0.1)	1.64 (0.1)	.54
Sex, n (%)			.62
Female	15 (68.1)	16 (72.7)	
Male	7 (31.9)	6 (27.3)	
Number of falls presurgery (within 1 year), mean (SD)	1.4 (0.6)	1.6 (0.7)	.88
NPRS^a^ (cm), mean (SD)	7.35 (1.1)	7.04 (1.2)	.72

a NPRS: Numeric Pain Rating Scale.

Table 2 shows inter-group comparisons of variables at baseline, 14th week, and 22nd week. The experimental group in this study demonstrated significant improvements across a range of outcome measures compared with the CG, reflecting substantial benefits in functional recovery and HRQOL. JPS showed superior accuracy in the experimental group (baseline [3.2 degrees] to 22 wk postsurgery [0.05 degrees]) compared with the CG (baseline [3.1 degrees] to 22 wk postsurgery [1.8 degrees]), with significant improvements noted (*P*=.001, Cohen *d*=3.1 vs 0.7). MSK-US measurements showed marked improvements in the experimental group (baseline [450.3 units] to 22 wk postsurgery [663.9 units]) compared with the CG (baseline [422.3 units] to 22 wk postsurgery [562.3 units]), indicating enhanced musculoskeletal health with a large effect size (Cohen *d*=3.7 vs 2.0, *P*=.001). Moreover, assessments using the BBS demonstrated significant gains in balance for the experimental group (baseline [34] to 22 wk postsurgery [53]) relative to the CG (baseline [37] to 22 wk postsurgery [48]), with substantial differences observed (*P*=.001, Cohen *d*=1.8 vs 0.4). The KOOS revealed consistent improvements across its various subscales (Pain, Symptoms, ADL, Sports/Recreation, QOL) for the experimental group, reflecting better knee function and QOL outcomes compared with the CG (all *P*=.001, Cohen *d* ranging from 1.6 to 4.9). Last, the EQ-5D-5L HRQOL scores were markedly higher for the experimental group (baseline [45.4] to 22 wk postsurgery [88.1]) compared with the CG (baseline [42.8] to 22 wk postsurgery [70.9]), indicating substantial gains in overall health status (*P*=.001, Cohen *d*=2.4 vs 1.3). Post-hoc correction for multiple comparisons using Holm-Bonferroni and Benjamini-Hochberg (FDR) methods was conducted, and all outcomes remained statistically significant after adjustment (*P*<.05) as shown in Multimedia Appendix 2. In conclusion, these findings exhibited greater improvements in the experimental group receiving supervised sensorimotor training with a lifestyle modification program than the CG, indicating potential benefits.

**Table 2.** Group-wise comparisons at baseline, 14 weeks postsurgery, and 22 weeks postsurgery.

Variable and group		Baseline (presurgery), mean (SD)	14 weeks postsurgery, mean (SD)	22 weeks postsurgery, mean (SD)	*P* value	*F* test (*df*)	Effect size	Cohen *d*
JPS^a^ (absolute error)					.001	7.1 (1, 42)	3.1	0.7
Intervention		3.2 (0.5)	1.8 (0.7)	0.05 (0.2)				
Control		3.1 (0.3)	2.1 (0.2)	1.8 (0.2)				
MSK-US^b^					.001	19.6 (1, 42)	3.7	2.0
Interventional		450.3 (52.4)	556.8 (53.6)	663.9 (60.2)				
Control		422.3 (51.5)	497.9 (48.5)	562.3 (42.9)				
BBS^c^					.001	9.6 (1, 42)	1.8	0.4
Interventional		34 (5)	48 (3)	53 (3)				
Control		37 (4)	45 (3)	48 (2)				
KOOS^d^								
KOOS (P)^e^					.001	19.0 (1, 42)	2.7	1.6
Interventional		38.3 (6.9)	55.3 (13.3)	81.2 (9.5)				
Control		32.3 (5.2)	61.0 (8.5)	66.9 (9.3)				
KOOS (S)^f^					.001	17.2 (1, 42)	3.8	1.3
Interventional		40.3 (9.0)	57.4 (10.2)	77.5 (7.0)				
Control		38.0 (13.4)	48.2 (11.8)	54.7 (10.3)				
KOOS (ADL)^g^					.001	11.2 (1, 42)	3.2	1.7
Interventional		57.6 (8.7)	65.0 (7.4)	70.8 (10.1)				
Control		60.1 (9.2)	66.3 (12.0)	67.5 (10.6)				
KOOS (SP)^h^					.001	12.6 (1, 42)	2.7	1.4
Interventional		26.6 (11.9)	35.7 (10.4)	44.5 (12.6)				
Control		30.3 (12.5)	38.0 (6.8)	40.2 (8.3)				
KOOS (QOL)^i^					.001	13.3 (1, 42)	4.9	2.6
Interventional		27.6 (8.1)	44.0 (11.7)	72.3 (9.8)				
Control		25.4 (9.6)	41.6 (10.8)	48.9 (8.5)				
EQ-5D-5L^j^					.001	8.4 (1, 42)	2.4	1.3
Interventional		45.4 (12.5)	68.2 (8.9)	88.1 (9.4)				
Control		42.8 (13.2)	64.6 (10.1)	70.9 (8.7)				

a JPS: joint position sense.

b MSK-US: musculoskeletal ultrasound.

c BBS: Berg Balance Scale.

d KOOS: Knee Injury and Osteoarthritis Outcome Score.

e KOOS (P): Knee Injury and Osteoarthritis Outcome Score (Pain).

f KOOS (S): Knee Injury and Osteoarthritis Outcome Score (Symptoms).

g KOOS (ADL): Knee Injury and Osteoarthritis Outcome Score (Activities of Daily Living).

h KOOS(SP): Knee Injury and Osteoarthritis Outcome Score (Sports).

i KOOS (QOL): Knee Injury and Osteoarthritis Outcome Score (Quality of life).

j EQ-5D-5L: EuroQol 5-Dimension 5-Level.

Table 3 demonstrates significant main effects and interaction effects across various outcome measures, indicating substantial differences between groups, over time, and the interaction of group and time.

**Table 3.** Main effects and interaction effects for outcome variables.

Dependent variables and source	Mean square	Partial Ƞ2	*P* value
JPS^a^			
Group	7.89	0.19	<.001
Time	4.56	0.12	<.001
Group * Time	6.78	0.18	<.001
MSK-US^b^			
Group	9.54	0.23	<.001
Time	5.67	0.16	.002
Group * Time	8.21	0.22	.001
BBS^c^			
Group	11.87	0.28	<.001
Time	6.78	0.18	.001
Group * Time	9.01	0.24	.001
KOOS^d^			
Group	9.32	0.22	.001
Time	5.12	0.14	.003
Group * Time	7.45	0.19	.001
EQ-5D-5L^e^			
Group	8.45	0.20	.001
Time	5.23	0.15	.002
Group * Time	7.12	0.19	<.001

a JPS: joint position sense.

b MSK-US: musculoskeletal ultrasound.

c BBS: Berg Balance Scale.

d KOOS: Knee Injury and Osteoarthritis Outcome Score.

e EQ-5D-5L: EuroQol 5-Dimension 5-Level.

JPS measurements showed significant main effects for the group (Mean square=7.89, Partial η²=0.19, *P*<.001) and time (mean square=4.56, Partial η²=0.12, *P*<.001), indicating improvements in JPS due to the intervention and over time. The interaction effect (Mean square=6.78, Partial η²=0.18, *P*<.001) supports that the experimental group had more significant gains in JPS.

MSK-US results showed significant main effects for the group (Mean square=9.54, Partial η²=0.23, *P*<.001) and time (Mean square=5.67, Partial η²=0.16, *P*=.002), suggesting significant improvements in musculoskeletal health due to the intervention and over time. The interaction effect (Mean square=8.21, Partial η²=0.22, *P*=.001) further supports that these improvements were more significant in the experimental group.

For balance, as measured by the BBS, there were significant main effects for the group (Mean square=11.87, Partial η²=0.28, *P*<.001) and time (Mean square=6.78, Partial η²=0.18, *P*=0.001), indicating substantial improvements in balance due to the intervention and over time. The interaction effect (Mean square=9.01, Partial η²=0.24, *P*=.001) highlights that the experimental group experienced more significant improvements in balance.

The KOOS results revealed significant main effects for the group (Mean square=9.32, Partial η²=0.22, *P*=.001) and time (Mean square=5.12, Partial η²=0.14, *P*=.003), indicating notable improvements in knee function and symptoms due to the intervention and over time. The interaction effect (Mean square=7.45, Partial η²=0.19, *P*=.001) suggests that these improvements were greater in the experimental group.

Finally, the EQ-5D-5L HRQOL scores showed significant main effects for the group (Mean square=8.45, Partial η²=0.20, *P*=.001) and time (Mean square=5.23, Partial η²=0.15, *P*=.002), demonstrating that the intervention significantly improved QOL and that these improvements were sustained over time. The interaction effect (Mean square=7.12, Partial η²=0.19, *P*<.001) indicates that the experimental group experienced more significant improvements in QOL.

Overall, these results demonstrate that the intervention had a substantial positive impact on functional mobility, pain reduction, musculoskeletal health, balance, knee function, JPS, and QOL. The significant interaction effects highlight that the experimental group benefited more from the intervention compared with the CG, with these benefits being sustained and increasing over time.

The telerehabilitation monitoring results demonstrated that self-compliance with the home-based lifestyle modification program varied between the intervention and CGs, with notable differences in adherence levels. Overall, 33 out of 44 participants (75%) exhibited more than 90% compliance with the program. Specifically, 18 out of 22 participants (81%) in the IG achieved over 90% compliance, compared with 15 out of 22 participants (68%) in the CG. Moderate compliance was observed in 10 out of 44 participants (23%) of the total sample, with 4 out of 22 participants (18%) of the IG and 6 out of 22 (27%) of the CG falling into this category. Only 1 out of 22 participants (4%) in the CG demonstrated low compliance, while the IG had no participants in this range. Importantly, no participants in either group had compliance levels below 51%, indicating that the majority were highly engaged with the program. These results suggest that the IG, supported by telerehabilitation monitoring, showed better adherence to the home-based lifestyle modification program compared with the CG, highlighting the potential benefits of telerehabilitation in enhancing compliance and engagement in post-TKA rehabilitation.

## Discussion

### Principal Findings

This study aimed to evaluate the effectiveness of supervised sensorimotor training with and without a lifestyle modification manual through telerehabilitation monitoring in patients undergoing TKA. The main findings indicate that both groups demonstrated improvements in pain, physical function, balance, joint proprioception, and QOL over time; however, the group receiving the combined intervention of sensorimotor training with lifestyle modification showed significantly greater improvements across almost all outcomes. Notably, enhancements were observed in the muscle thickness (MSK-US), balance (BBS), and all subscales of the KOOS, as well as JPS and HRQOL (EQ-5D-5L). The CG did not receive a structured lifestyle plan emphasizing exercise, diet, and education instructions via a web portal; instead, they were instructed to maintain their usual daily routine. Consistent communication was maintained through the telerehabilitation mode, providing continuous guidance and exercise instructions. Both groups received telerehabilitation monitoring, but the lifestyle modification plan was provided exclusively to the IG. Both groups demonstrated improvement in various outcome measures; however, the experimental group had superior outcomes. Specifically, the experimental group showed greater improvements in functional mobility, pain reduction, musculoskeletal health, balance, knee function, JPS, and overall QOL compared with the CG.

Participants in the experimental group, who received the lifestyle modification plan, showed significantly greater improvements in knee function as measured by the KOOS. The improvements spanned multiple subscales, including Pain, Symptoms, ADL, Sports/Recreation, and QOL. This aligns with previous studies demonstrating the efficacy of comprehensive rehabilitation programs in enhancing knee function postsurgery [28]. However, the addition of lifestyle modifications appears to amplify these benefits, suggesting that addressing behavioral and lifestyle factors plays a crucial role in optimizing recovery [29]. The significant increase in the musculoskeletal cross-sectional area, as measured by MSK-US, further supports the advantage of the combined approach. The experimental group exhibited greater increases in cross-sectional area compared with the CG, indicating enhanced musculoskeletal health and strength. This finding contrasts with earlier studies that focused solely on exercise interventions, which reported modest improvements in muscle mass and strength [30]. The inclusion of lifestyle modifications likely contributed to the observed gains by promoting overall physical health and activity levels. JPS, a critical component of proprioception, showed marked improvements in the experimental group. The reduction in absolute error in JPS was significantly greater for participants receiving lifestyle modifications, suggesting that this comprehensive approach enhances proprioceptive accuracy. Previous research has highlighted the benefits of sensorimotor training in improving proprioception post-TKR. This study extends these findings by demonstrating that lifestyle modifications can further refine proprioceptive function [31]. Similarly, balance, as measured by the BBS, improved more significantly in the experimental group. The substantial gains in balance scores indicate that lifestyle modifications, in conjunction with sensorimotor training, provide a more holistic approach to rehabilitation [32]. These findings are consistent with previous literature that emphasizes the role of balance training in improving postural stability postsurgery [33]. However, this study suggests that incorporating lifestyle changes may lead to even greater improvements in balance and stability.

Pain reduction, as measured by the Numeric Pain Rating Scale, was significantly more pronounced in the experimental group. Participants reported lower pain levels throughout the study period, underscoring the effectiveness of the combined intervention in managing postsurgical pain. This aligns with existing studies that have shown exercise and rehabilitation programs can reduce pain levels [34]. The additional benefit observed in this study may be attributed to lifestyle modifications that address inflammation, diet, and overall well-being, contributing to pain reduction [35]. QOL, assessed using the EQ-5D-5L, showed substantial improvements in the experimental group. The significant gains in HRQOL scores indicate that the combined approach not only enhances physical recovery but also positively impacts overall well-being and satisfaction [36]. These findings resonate with previous research that has demonstrated the positive impact of comprehensive rehabilitation programs on QOL post-TKR [37]. The added value of lifestyle modifications highlights the importance of addressing holistic health factors in rehabilitation programs.

The telerehabilitation monitoring results demonstrated higher adherence rates in the experimental group, suggesting that lifestyle modifications may enhance engagement and compliance with rehabilitation programs [34]. The majority of participants in the experimental group achieved over 90% compliance, indicating that the combined approach was well-received and effectively implemented. These findings are in line with studies emphasizing the role of telerehabilitation in supporting adherence to home-based rehabilitation programs [38]. This study adds to this body of evidence by showing that lifestyle modifications, supported by telerehabilitation, can further improve adherence and outcomes.

To our knowledge, this is the first study of lifestyle modification programs conducted among TKA patients and using the telerehabilitation monitoring method in Pakistan. A noteworthy feature of this study is the translation of the lifestyle modification manual into Urdu, the native language of the Pakistani population. This translation ensured that participants fully understood the lifestyle recommendations and guidelines, thereby enhancing the effectiveness of the intervention. The translation addressed language barriers and made the program more accessible and culturally relevant to the participants, contributing to the high compliance and positive outcomes observed in the experimental group.

However, the study also has certain limitations. The use of a single clinical care ensured superior standardization of care, which can be regarded as a notable benefit in the context of conducting this exploratory and innovative trial. This standardization allowed for consistent and controlled conditions, thereby enhancing the reliability of the results. Nevertheless, the capacity to generalize the findings from this study to other settings, joint systems, and surgical procedures is expected to be limited in comparison to results derived from more diverse multicenter trials. An additional limitation of this innovative study is that, although it was hypothesized that the lifestyle program would enhance participants’ confidence to safely engage in a more physically active lifestyle, physical activity was not directly assessed. Future studies should also focus on assessing the cost-effectiveness of telerehabilitation, predominantly in the framework of developing nations like Pakistan. Evaluating economic feasibility, possible savings, and application barriers could benefit in larger-scale adoption.

### Conclusions

Both groups that received sensorimotor training demonstrated improvements in their outcomes. However, the incorporation of a lifestyle modification program in the experimental group yielded significantly better results compared with those who received the standard intervention alone. The experimental group showed superior improvements in knee function, performance, and overall QOL. These findings suggest that integrating a lifestyle modification program with sensorimotor training can enhance rehabilitation outcomes for patients undergoing total knee replacement. In addition, the IG, supported by telerehabilitation monitoring, showed better adherence to the home-based lifestyle modification program compared with the CG, highlighting the potential benefits of telerehabilitation in enhancing compliance and engagement in lifestyle modification rehabilitation programs.

## Supplementary material

10.2196/64643 Multimedia Appendix 1 Lifestyle modification plan.

10.2196/64643 Multimedia Appendix 2 Post-hoc adjusted P values.

10.2196/64643 Checklist 1 CONSORT eHealth checklist.

## Abbreviations

ADL: activities of daily living
BBS: Berg Balance Scale
CG: control group
EQ-5D-5L: EuroQol 5-dimension 5-level questionnaire
HRQOL: health-related quality of life
IG: intervention group
JPS: joint position sense
KOOS: Knee Injury and Osteoarthritis Outcome Score
MSK-US: musculoskeletal ultrasound
OA: osteoarthritis
QOL: quality of life
RF: rectus femoris
TKA: total knee arthroplasty
VAS: Visual Analog Scale
