# Supplementary material for: Effect of Lifestyle Modification Through Web-Based Telerehabilitation Monitoring Combined With Supervised Sensorimotor Training After Total Knee Arthroplasty: Randomized Controlled Trial
Source: JMIR Mhealth Uhealth. 2025 Oct 2;13:e64643. doi: 10.2196/64643 (PMC12490816; doi:10.2196/64643)
Supplement: Multimedia Appendix 1 [file mhealth-v13-e64643-s001.pdf]

گھٹنے کی مکمل تبدیلی کے لیے طرز زندگی میں بدلاؤ کا دستور العمل

ورزش

صبح کی ورزشیں

ورزش	ہدایات	تفصیلات	مطلوبہ تعداد / دہرانا
ران کے پچھلے حصے کے پٹھوں کو کھینچنا	دروازے کے قریب اپنی پیٹھ کے بل لیٹتے ہوئے، آپریشن والی ٹانگ کو دروازے سے باہر اور دوسری ٹانگ کو دروازے کے فریم کے ساتھ اوپر رکھیں۔ اپنی ران کے پچھلے حصے میں درمیانے درجے کا کھنچاؤ محسوس کریں۔	دیوار	1منٹ
پنڈلی کے پٹھے کو کھینچنا	دونوں بازو دیوار پر رکھ کر دیوار کے سامنے کھڑے ہوں، اپنی آپریشن والی ٹانگ کے سامنے اپنی دوسری ٹانگ رکھیں اور اپنی ٹانگ کے نچلے حصے کی پچھلی جانب موجود پنڈلی کے پٹھے کو کھینچنے کے لیے دیوار کے ساتھ ٹیک لگالیں۔	دیوار	1منٹ
ران کے اگلے حصے کے پٹھوں کو کھینچنا	دیوار کے ساتھ پیٹھ لگا کر کھڑے ہوتے ہوئے اپنی آپریشن والی ٹانگ کے پاؤں یا ٹخنے کو پکڑیں اور آہستگی سے پیچھے کی طرف کھینچیں۔ سہارے کے لیے دوسرے ہاتھ سے کرسی کو پکڑ لیں۔ آپ کو اپنی ران کے اگلے حصے میں کھنچاؤ محسوس ہونا چاہیے۔ آپ یہ کھنچاؤ پیٹ کے بل لیٹ کر اور گھٹنے پیچھے موڑ کر اپنے ہاتھ یا تولیے سے پکڑ کر اس حالت میں بھی کر سکتے ہیں۔ اپنی ران کے اگلے حصے میں درمیانے درجے کا کھنچاؤ محسوس کریں۔	تولیہ، دروازے کا فریم	2منٹ

دوپہر کی ورزشیں

ورزش	ہدایات	تفصیلات	مطلوبہ تعداد / دہرانا
6انچ اوپر آگے کو بڑھنا	6انچ کی بلندی پر اوپر آگے اور پھر پیچھے نیچے کی طرف قدم بڑھائیں۔	6انچ اونچی 2 بڑی کتابیں یا 6 انچ اونچا بڑا کارڈ بورڈ	10/2
سہارے کے ساتھ ایک قدم پر ران کے پٹھے سے ایک ٹانگ کو اٹھانا	اپنی آپریشن والی ٹانگ کو سہارے کے ساتھ آہستہ آہستہ پنجوں کے بل لائیں اور پھر قدم کی سطح سے نیچے لے جائیں، تاکہ آپ اپنے ران کے پٹھے کو کھنچتے ہوئے محسوس کر سکیں۔	اوپر چڑھنا یا آگے بڑھنا	10/1
ایک سائیڈ کو 2انچ قدم اٹھانا	اپنی آپریشن والی ٹانگ کو سیڑھی پر اور اپنی دوسری ٹانگ کو ایک طرف زمین پر رکھتے ہوئے، اپنا سارا وزن آپریشن والی ٹانگ پر منتقل کریں اور آہستہ آہستہ اپنی دوسری ٹانگ کو فرش سے اٹھائیں، سیڑھی کو چھوئیں اور آہستہ آہستہ ابتدائی حالت پر نیچے لے جائیں۔	2انچ کی 1-2 کتابیں	10/2
باری باری ٹانگوں کو آگے پیچھے کرنا	ایک قدم کو آگے رکھیں اور آگے نیچے کو جھکیں، اور پھر اگلی ٹانگ کو واپس پہلی حالت پر لاکر دوسری ٹانگ کے ساتھ یہی عمل دہرائیں۔	کوئی نہیں	10/1

★ National University of Modern Languages Islamabad ★
Department of Translation and Interpretation
Date 13/2/2023

دیوار کے سہارے بیٹھنا	اپنی پیٹھ کو دیوار پر ٹکا کر کھڑے ہو جائیں اور پھر دونوں گھٹنوں کو 90 درجے تک جھکائیں اور کندھے کی چوڑائی کو الگ رکھیں۔ اگر آپ کو لگتا ہے کہ آپ گرنے والے ہیں تو سہارے کے لیے قریب کوئی چیز رکھ لیں، تاکہ جب آپ واپس سیدھی حالت پر آئیں تو وہ آپ کے لیے مددگار ہو۔	دیوار	1 منٹ
گھٹنے کے پچھلے پٹھوں کو موڑ کر کھڑے ہونا	کیبل مشین یا مزاحمتی رسی کا استعمال کرتے ہوئے 1-5 پاؤنڈ مزاحمت کے ساتھ بغیر درد کے اپنے گھٹنے کو موڑ کر کھڑے ہونا۔	کیبل مشین یا مزاحمتی رسی	10/1
گھٹنے کو موڑ کر کھڑے ہونا	کیبل مشین یا مزاحمتی رسی کا استعمال کرتے ہوئے 1-5 پاؤنڈ مزاحمت کے ساتھ بغیر درد کے اپنے گھٹنے کو موڑ کر کھڑے ہونا۔	کیبل مشین یا مزاحمتی رسی	10/1
ایک ٹانگ پر اٹھنا بیٹھنا	ایک مضبوط کرسی کا استعمال کرتے ہوئے اپنے کولہے کو نیچے کی طرف لائیں اور کرسی پر بیٹھ جائیں۔ پھر اپنی ٹانگ کے پٹھوں کا استعمال کرتے ہوئے بازو کا سہارا لیے بغیر آہستہ آہستہ اٹھیں۔	کرسی	10/1
سیدھی ٹانگ ٹخنے پر وزن کے ساتھ 16 انچ تک اوپر اٹھانا	پیٹھ کے بل لیٹ جائیں اپنے گھٹنے کے اوپر والے پٹھوں کو اکڑائیں اور اپنی پوری ٹانگ کو فرش سے 1 انچ اٹھائیں، صرف اس صورت میں جبکہ آپ اپنی ٹانگ اٹھاتے وقت گھٹنے کو موڑنے کے بغیر ایسا کر سکتے ہوں۔ اپنی برداشت کے مطابق 2-5 پاؤنڈ کے درمیان ٹخنے پر وزن شامل کریں۔	کوئی نہیں	10/1

شام کی ورزشیں

ٹخنے پر وزن کے ساتھ سامنے پھیلا ہوا گھٹنا موڑنا	پیٹ کے بل لیٹتے ہوئے اپنے گھٹنے کو سیدھے اوپر سے مکمل موڑنے تک اور پھر نیچے کی طرف موڑیں۔ 2-5 پاؤنڈ ٹخنے پر وزن استعمال کریں۔ حرکت آپ کے آپریشن والے گھٹنے کے برابر نہ ہو تو اس کے بہت قریب ہونی چاہیے۔	ٹخنے پر وزن: 2-5 پونڈ	10/1
سہارا لے کر ایک ٹانگ کو آدھا نیچے بٹھانا	اپنا پورا وزن آپریشن والی ٹانگ پر ڈال کر اور دوسرے گھٹنے کو موڑ کر اپنے جسم کو آہستہ آہستہ نیچے لائیں یہاں تک کہ آپ کے گھٹنے 60 درجے تک جھک جائیں۔ کچھ سہارا لینے کے لیے کسی مضبوط کرسی کی پشت کا استعمال کریں۔ جب آپ اپنے جسم کو نیچے رکھیں تو اپنے کولہے کو باہر رکھیں اور اگر آپ کو سہارے کی ضرورت ہو تو قریب ایک میز رکھ لیں۔	کرسی، میز	10/1
ایک ٹانگ پر پل بنانا	پشت کے بل لیٹے ہوئے اپنے آپریشن والے گھٹنے کو جھکا کر اور دوسرے گھٹنے کو سیدھا رکھ کر صرف اپنے آپریشن والے پاؤں کے ذریعے نیچے کی طرف وزن ڈالیں اور اپنے کولہوں کو فرش سے اٹھائیں جب تک کہ آپ کے کولہے سیدھے نہ ہو جائیں اور پھر آہستہ آہستہ نیچے لائیں۔	درکار سامان: کوئی نہیں۔	10/2
10 فٹ تک پاؤں ایک دوسرے کے آگے رکھ کر توازن سے چلنا	10 فٹ کے فاصلے پر 2 ہدف متعین کریں۔ آگے جاتے ہوئے اپنی دائیں ٹانگ کو بائیں ٹانگ کے اور پھر آگے جاتے ہوئے بائیں ٹانگ کو دائیں ٹانگ کے آگے رکھیں۔ پھر اسی عمل کو پیچھے کی طرف دہرائیں۔ جیسے جیسے آپ بہتر ہوتے جائیں اپنی رفتار بڑھاتے جائیں۔	2 اہداف	5 منٹ
توازن کے ساتھ آگے پیچھے چلنا	5،10،15 فٹ کے فاصلے پر 3 اہداف متعین کریں۔ 5 فٹ چلنے سے آغاز کریں اور پھر سٹارٹ پوائنٹ تک پیچھے کی طرف چلیں، پھر 10 فٹ آگے چلیں اور 5 فٹ پیچھے چلیں اور پھر 15 فٹ کے ہدف کی طرف آگے چلیں اور 10 فٹ والے ہدف کی طرف پیچھے کی طرف چلیں اور پھر 15 فٹ کے ہدف تک چل کر ختم کریں۔ جیسے جیسے اس ورزش میں بہتری محسوس کریں تو رفتار بڑھاتے جائیں۔	3 اہداف	5 منٹ
سمتوں کو تبدیل کرنے میں توازن	اپنے ساتھی کی طرف منہ کر کے کھڑے ہوں اور وہ آپ کو ہاتھ سے آگے، پیچھے، دائیں، بائیں حرکت کرنے کا اشارہ کرے۔ ایک بار جب آپ کا ساتھی اشارہ کر دے تو اگلا اشارہ دینے تک اسی سمت میں بڑھتے رہیں۔ 30 سیکنڈ کے مقابلے کل 5 منٹ تک جاری رکھیں۔	ایک اور شخص کی ضرورت ہے	5 منٹ

★ National University of Modern Languages Islamabad ★
Department of Translation and Interpretation
Date 13/2/2023

ران کے پچھلے حصے کے پٹھوں کو کھینچنا	دروازے کے قریب اپنی پیٹھ کے بل لیٹتے ہوئے، آپریشن والی ٹانگ کو دروازے سے باہر اور دوسری ٹانگ کو دروازے کے فریم کے ساتھ اوپر رکھیں۔ اپنی ران کے پچھلے حصے میں درمیانے درجے کا کھنچاؤ محسوس کریں۔	دیوار	1منٹ
پنڈلی کے پٹھے کو کھینچنا	دونوں بازو دیوار پر رکھ کر دیوار کے سامنے کھڑے ہوں، اپنی آپریشن والی ٹانگ کے سامنے اپنی دوسری ٹانگ رکھیں اور اپنی ٹانگ کے نچلے حصے کی پچھلی جانب موجود پنڈلی کے پٹھے کو کھینچنے کے لیے دیوار کے ساتھ ٹیک لگالیں۔	دیوار	1منٹ
ران کے اگلے حصے کے پٹھوں کو کھینچنا	دیوار کے ساتھ پیٹھ لگا کر کھڑے ہوتے ہوئے اپنی آپریشن والی ٹانگ کے پاؤں یا ٹخنے کو پکڑیں اور آہستگی سے پیچھے کی طرف کھینچیں۔ آپ کو اپنی ران کے اگلے حصے میں کھنچاؤ محسوس ہونا چاہئے۔<br>آپ یہ کھنچاؤ پیٹ کے بل لیٹ کر اور گھٹنے پیچھے موڑ کر اپنے ہاتھ یا تولیے سے پکڑ کر اس حالت میں بھی کر سکتے ہیں۔ اپنی ران کے اگلے حصے میں درمیانے درجے کا کھنچاؤ محسوس کریں۔	تولیہ، دروازے کا فریم	2منٹ

تعلیم

چیک لسٹ	ہدایات
درد سے بچیں	اس موقع پر آپ کو محدود سا درد ہونا چاہیے۔ اگر آپ کو اب بھی بہت زیادہ درد ہو تو فوری طور پر اپنے صحت کی سہولت فراہم کرنے والے سے رجوع کریں۔
سوجن سے بچیں	آپ کو اس وقت سوجن نہیں ہونی چاہیے یا بہت معمولی ہونی چاہیے۔ اگر آپ کو اب بھی بہت زیادہ درد ہو تو فوری طور پر اپنے صحت کی سہولت فراہم کرنے والے سے رجوع کریں۔
توازن کی ہدایات	گھٹنے کی تبدیلی کے بعد توازن کی مشق ضروری ہے۔ آپ کونسی ورزشیں کر سکتے ہیں، اس کا دارومدار اس بات پر ہے کہ آپ سرجری سے پہلے کیا کر سکتے تھے۔ ہر ایک ورزش صرف اس صورت میں کریں جب آپ کو گرنے یا چوٹ لگنے کا کوئی خطرہ نہ ہو۔ یہ یقینی بنانے کے لیے کہ آیا آپ ان ورزشوں کو مکمل کرنے کے لیے محفوظ ہیں اپنے معالج سے مشورہ کریں۔
کھنچاؤ کی معلومات	کھنچاؤ کے لیے ضروری ہے کہ آپ 10 سیکنڈ تک ایک حالت پر قائم رہیں اور درمیانے درجے کے کھنچاؤ کا احساس برقرار رکھیں۔ اگر کھنچاؤ بہت زیادہ محسوس ہوتا ہے تو اس سے کوئی فائدہ نہیں ہوگا۔ اس کے علاوہ کھنچاؤ کی حالت کو بار بار تبدیل نہ کریں۔
تیز چلنا	ہفتے میں 4 بار 30 منٹ کے لئے مضبوط ہموار سطح پر تیز چلیں۔
گھٹنے کی حرکت کی طاقت کو محفوظ رکھیں	اگر آپ کے معالج نے آپ کو ایسا کرنے کی ہدایت کی ہے یا اگر آپ کا گھٹنا مکمل حرکت کرنے نہیں لگا ہے تو، گھٹنے کو موڑنے اور سیدھا کرنے کی مشقیں جاری رکھیں۔ ورنہ ان کی ضرورت نہیں ہے۔

ورزشی سائیکل کے ساتھ متبادل تیز واک

ورزشی سائیکل	سیٹ نیچے اور درمیانی سختی والی ورزشی سائیکل پورے چکر کے ساتھ ہفتے میں 3 بار	ورزشی سائیکل	30منٹ

غذائیت (خوراک کا چارٹ)

* National University of Modern Languages Islamabad
Department of Translation and Interpretation *
Date 13/2/2023

**ناشتہ**	ناشتے سے پہلے پانی یا لیموں پانی (نیم گرم پانی میں لیموں) پی لیں۔<br>پھر<br>جو کی روٹی کے ساتھ 1 انڈا (ترجیحی طور پر ابلا ہوا)<br>ساتھ<br>چائے / دودھ (کم چکنائی والا دودھ)<br>یا<br>دودھ والا جو کا دلیہ
**وسطی صبح**	اپنی پسند کے مطابق 1 پھل یا<br>تازہ رس (پیک شدہ نہیں) یا<br>½ کپ تازہ بیریز یا<br>پھلوں کا شیک (وزن کم کرنے کے لیے سب سے زیادہ قابل ترجیح) (اس میں کم چکنائی والا دہی استعمال کریں)
**دوپہر کا کھانا**	1 کپ مکس سلاد (کھیرا، ٹماٹر، گوبھی شامل کریں پھر ذائقہ کے لیے ذرا سا لیموں کا رس اور کالی مرچ ڈال لیں)<br>+<br>3 اونس بھُنا ہوا دیسی مرغی مچھلی / گوشت<br>یا<br>½ کپ سبزیوں کا سالن / دال 1 روٹی کے ساتھ۔ (پالک وزن کم کرنے میں مدد گار ہے) بین سلاد کے ساتھ<br>دوپہر کے کھانے میں کم چکنائی والا دہی بھی شامل کریں (اگر آپ پہلے فروٹ شیک پی چکے ہیں تو دوپہر کے کھانے میں ضروری نہیں ہے)<br><br>#اگر آپ چاول لینا چاہتے ہیں تو آپ سالن کے ساتھ ½ کپ ابلے ہوئے چاول لے سکتے ہیں۔
**شام**	اگر پسند کرتے ہیں تو چائے<br>1 یا 2 بسکٹ کے ساتھ<br>یا<br>سبز چائے (دار چینی اور لیموں شامل کریں اور چینی کی بجائے شہد استعمال کریں)
**رات کا کھانا**	بین سلاد (لوبیا، چنے، مٹر)<br>½ سبزی یا کسی دال کا سالن چھوٹی سی روٹی کے ساتھ۔
**سونے کے وقت**	1 گلاس دودھ (صرف کم چکنائی والا)<br>یا<br>بادام

**وہ کھانے جن کی اجازت ہے**	**وہ کھانے جن سے پرہیز کرنا ہے**
تازہ سبزیاں	شراب
جو کا دلیہ	زیادہ میٹھے والے کھانے
کم چکنائی والی دودھ کی مصنوعات	تلی ہوئی اشیاء

★ National University of Modern Languages Islamabad ★
Department of Translation and Interpretation
Date 13/2/2023

موانع تکسید	پروسیس شدہ کھانے
لیموں پانی	زیادہ چکنائی
گری دار میوے، زیادہ تر بادام	برائلر چکن
تازہ پھل	زیادہ چکنائی والا کھانا/ڈیری مصنوعات

★ National University of Modern Languages Islamabad ★
Department of Translation and Interpretation
Date 13/2/2023
