# Supplementary material for: Effect of Lifestyle Modification Through Web-Based Telerehabilitation Monitoring Combined With Supervised Sensorimotor Training After Total Knee Arthroplasty: Randomized Controlled Trial
Source: JMIR Mhealth Uhealth. 2025 Oct 2;13:e64643. doi: 10.2196/64643 (PMC12490816; doi:10.2196/64643)
Supplement: Multimedia Appendix 2 [file mhealth-v13-e64643-s002.docx]

| **Outcome Measure** | **Raw P-Value** | **^a^Holm-Bonferroni Adjusted P** | **^b^Benjamini-Hochberg Adjusted P** |
| --- | --- | --- | --- |
| MSK-US | .001 | .009 | .001 |
| BBS | .001 | .009 | .001 |
| KOOS (Pain) | .001 | .009 | .001 |
| KOOS (Symptoms) | .001 | .009 | .001 |
| KOOS (ADL) | .001 | .009 | .001 |
| KOOS (Sports) | .001 | .009 | .001 |
| KOOS (QoL) | .001 | .009 | .001 |
| JPS (Absolute Error) | .001 | .009 | .001 |
| EQ 5D 5L | .001 | .009 | .001 |

^a^Holm-Bonferroni correction applied for 9 comparisons (adjusted p = .009).
^b^ Benjamini-Hochberg FDR correction applied for 9 comparisons (adjusted p = .001).
